# Items

**Published:** 1908-01

**Authors:** 


### ITEMS.

The Denver Chemical Manufacturing Company, makers and distributors of Antiphlogistine, has sent out a leather banknote and card case, together with a fully paid accident policy for

$2.500, in token of their success in the past and excellent prospects for the future. The insurance policy is a novel but generous way of recognising their aid received from medical journals.

---

Battle & Company, Saint Louis, has issued number 4 of the series of eighteen illustrations of dislocations. This number presents an etching of downward and forward dislocation of the sternal end of the clavicle.

---

Public Health and Marine Hospital Service Examinations.—A board of commissioned medical officers will be convened to meet at the Bureau of Public Health and Marine-Hospital Service, 3 B street SE., Washington, D. C., Monday, January 20, 1908, at 10 o'clock a.m., for the purpose of examining candidates for admission to the grade of assistant surgeon in the Public Health and Marine-Hospital Service. Candidates must be between 22 and 30 years of age, graduates of a reputable medical college, and must furnish testimonials from responsible persons as to their professional and moral character. The following is the usual order of the examinations: 1, physical; 2, oral; 3, written; 4, clinical. The tenure of office is permanent. For further information, or for invitation to appear before the board of examiners, address Surgeon-General, Public Health and Marine-Hospital Service, Washington, D. C.

---

M. J. Breitenbach Company, 53 Warren Street, New York, proprietors and distributors of Pepto-Mangan (Gude), that excellent form of iron and manganese for the anemias, have again sent out to the profession the Physicians' Daily Memorandum, this edition being for the year 1908. It is one of the most serviceable desk memorandum books with which we are familiar, one page being allotted to each day of the year.

---

The United States Civil Service Commission announces an examination on January 15, 1908, to secure eligibles from which to make certification to fill vacancies as they may occur in the position of physician (male) in the Indian Service, at salaries ranging from $720 to $1 200 per annum. Applicants should at once apply either to the United States Civil Service Commission, Washington, D. C., or to the secretary of the board of examiners at Buffalo, for application Form 1312. No application will be accepted unless properly executed and filed with the Commission at Washington.

